# Supplementary material for: Dominant-negative STAT5B mutations cause growth hormone insensitivity with short stature and mild immune dysregulation
Source: Nat Commun. 2018 May 29;9:2105. doi: 10.1038/s41467-018-04521-0 (PMC5974024; doi:10.1038/s41467-018-04521-0)
Supplement: Supplementary file 1 — Supplementary Information [file 41467_2018_4521_MOESM1_ESM.pdf]

Supplementary Information

Dominant-negative STAT5B mutations cause growth hormone insensitivity with short stature and mild immune dysregulation

Klammt et al.

Supplementary information

List of contents

Supplementary Note 1. Detailed patient reports p. 1
Supplementary Table 1. Extended IGF1 generation test family 2 p. 8
Supplementary Table 2. Penetrance of major clinical characteristics p. 8
Supplementary Table 3. WES, compound heterozygous candidate variants, family 1 p. 9
Supplementary Table 4. WES, dominant candidate variants, families 2 and 3 p. 9
Supplementary Table 5. *in silico* analysis of STAT5B variants p. 10
Supplementary Table 6. Concordance rates of variants between family members, family 1 p. 10
Supplementary Figure 1. STAT alignment and 3D models p. 11
Supplementary Figure 2. Comparison height SDS of GHIS conditions p. 13
Supplementary Figure 3. Microcephaly is a not a common feature in AD STAT5B patients p. 14
Supplementary Figure 4. Uncropped captures of immunoblots (Fig. 2a and b) p. 15
Supplementary Figure 5. Uncropped captures of immunoblots (Fig. 2c) p. 16
Supplementary References p. 17

Supplementary Note 1. Detailed patient reports

Family 1. The Caucasian boy (Proband 1; Fig. 1a in the main text; subject II-2, Family 1), one of two monozygotic twins, was born to a non-consanguineous Caucasian couple after an uneventful pregnancy at the 36th gestational week with a birth weight of 2,500 g (standard deviation score, SDS -0.9) and a birth length of 45 cm (-1.7 SDS). At 12 months he had pneumonia with hospital admission, followed by several attacks of bronchitis within the next 3 years. Otherwise, his postnatal period was unremarkable with a slight developmental delay. Starting from 4 years of life he was treated with inhaled corticosteroids and long-acting beta 2-agonists for moderate but persistent bronchial asthma. Since his neonatal period he had severe, periorbital eczema.

At first endocrine evaluation of severe growth delay at the age of 14.5 years, he presented with proportionate short stature with a height of 131.5 cm (-5.3 SDS) and weight of 28 kg (-4.5 SDS). No dysmorphic signs were apparent. He was healthy and performed well in school. His IGF1 serum concentration was at the lower detection limit, while basal GH and response to a clonidine stimulation test were normal indicating insensitivity to growth hormone (Table 1). His bone age (Tanner-Whitehouse 3 method[1]) was retarded by approximately 5 years and puberty had not started (Tanner stage P1G1). Recombinant hIGF1 therapy [0.12 mg kg^{-1} twice daily] was initiated. At 16.2 years he measured 142 cm (-5.3 SDS) and after 3 years of rhIGF1 treatment at the age of 17.8 years his height remained low at -4.4 SDS (153.1 cm) although growth was still ongoing. Head circumference was 56 cm (-0.53 SDS). At that age, development of secondary pubertal signs had advanced considerably (Tanner stage P4G4), yet bone age was still retarded by about 4 years. Endocrine evaluation at 16.5 years confirmed barely

detectable IGF1 serum concentrations and low acid-labile subunit (IGFALS), whereas IGFBP3, prolactin and growth hormone-binding protein (GHBP) concentrations were in the normal or low-normal range (Table 1). Repeated immunological profiles evaluations were not suggestive for immunodeficiency. However, immunoglobulin E (IgE) was in the upper range of normal (156 kU l^{-1}, normal: < 200 kU l^{-1}) or elevated (340 kU l^{-1}, normal: < 114 kU l^{-1}).

The index patient's twin brother (Family 1, II-3) presented with nearly identical clinical, endocrine, and immunological features (Supplementary Table 2). His IgE values were consistently above the normal range (228 kU l^{-1} and 382 kU l^{-1} at 2 different evaluations), thus corroborating the respective findings in the index patient. Of note, rhIGF1 treatment, initiated at the same age as for his brother, was discontinued at the age of 17.1 years. Monitoring his growth pattern in the follow-up did not reveal any substantial difference compared to his twin brother, for whom therapy continued. Father's and mother's height were normal (174.5 cm, -0.9 SDS and 159.4 cm, -1.2 SDS, respectively). The father entered puberty late with a height of 155 cm at the age of 16 years. The twins have an older brother and a half-sister from father's second partnership (Fig. 1a), who are all reported to be healthy and of normal height.

Targeted molecular genetic analysis revealed a heterozygous variant (c.530A>C) in exon 5 of the *STAT5B* gene that was found in both twins but not in the parents. The *de novo* mutation is predicted to result in a substitution of proline for glutamine (p.Gln177Pro) within α-helix 1 of the coiled-coil domain of the STAT5B protein, presumably disrupting the secondary structure of the helix. Analyses of the *GHR*, *GH1*, *IGF1*, *IGFALS*, and *IGF1R* genes did not disclose any further potentially pathogenic aberration. Exon-spanning, overlapping RT-PCR amplifications covering the entire *STAT5B* mRNA confirmed expression of both alleles and excluded any additional anomaly in the wild-type allele that might have escaped Sanger sequencing of genomic DNA.

WES analysis was performed with DNA samples of the twins and their parents. As there are two affected brothers with unaffected parents and an unaffected sibling, the genetic pattern of inheritance could be consistent with either a *de novo* dominant, homozygous recessive, compound heterozygous, or X-linked form of inheritance. Analysis pipelines for the homozygous recessive and the X-linked recessive model yielded no variants that met the filter criteria. There were three genes that met the definition of the compound heterozygous model: *SSUH2*, *BSN*, *DNHD1* (Supplementary Table 3). In each of these genes, one of the variants was predicted to be benign by either Polyphen2 or SIFT decreasing the likelihood that these variants are causal. Furthermore, none of these genes were good biological candidates. Testing of the *de novo* dominant model revealed only a single variant that met the filter criteria – the c.530A>C, p.Gln177Pro, variant in *STAT5B*.

From WES data, the variant was further confirmed to be *de novo* by calculating the concordance rates between single nucleotide variants amongst the family members (Supplementary Table 6), which demonstrated that each affected child was confirmed to have ~50% concordance with each parent which is the expected rate for a parent-child dyad. Additionally, the siblings were confirmed to have >98% concordance rate for single nucleotide variants that pass filters, which further confirms that they are in fact

identical twins. The small percentage of discordant calls is attributable to sequence coverage issues as well as sequencing artifacts.

Family 2. Proband 2 (Fig. 1a, subject III-1, Family 2) is the eldest son of a non-consanguineous Caucasian couple. He was born at term after a normal pregnancy by emergency caesarian section for fetal distress. His birth weight was 3,460 g (0.1 SDS). There were no neonatal problems. He was bottle-fed and had normal developmental milestones. He was a fussy eater and was noted to have a high squeaky voice. He had eczema since the age of 2 weeks for which he received 1% hydrocortisone cream and emollient creams and which improved over time. He had uncomplicated chickenpox at the age of 3 years. Since his earliest height measurement at the age of 1.9 years his growth had followed the -2.9 SDS line. Celiac disease was excluded. IGF1 concentrations were below the detection limit despite a normal GH provocation test at that age (baseline GH 3.2 ng ml^{-1}, peak GH 17.3 ng ml^{-1}; Table 1). Overnight GH sampling at the age of 3 showed normal peaks of up to 12.4 ng ml^{-1} with detectable GH in between peaks, suggestive of mildly increased GH production. A 4-day IGF1 generation test did not show a rise in IGF1 concentration. Since growth hormone insensitivity was suspected, an extended IGF1 generation test was performed at the age of 5 years with a rhGH response of 83 ng ml^{-1} IGF1 (normal range: 57 – 316 ng ml^{-1}) only at the highest GH dose (2.8 mg m^{-2} d^{-1}) confirming GH insensitivity (Supplementary Table 1)[2]. IGFBP3 remained within the low-normal range. Prolactin, free thyroxine (fT4), and cortisol were normal, and thyroid-stimulating hormone (TSH) was slightly elevated (6.4 mU l^{-1}, normal: < 6 mU l^{-1}). Magnetic resonance imaging of the pituitary was normal. Immunological evaluation revealed no abnormality except elevated IgE concentrations of 118 kU l^{-1} (normal: < 52 kU l^{-1}). At the age of 8.6 years his height was -3.0 SDS (114.0 cm), HC measured at 9.4 years was 51.6 cm (-1.70 SDS). His bone age (Tanner-Whitehouse 2 method) was delayed at 6.1 years. IGF1 and IGFBP3 continued to be low [IGF1, 35 ng ml^{-1} (74-388 ng ml^{-1}); IGFBP3, 1.51 mg l^{-1} (1.8-7.1 mg l^{-1})]. Prolactin was normal [350 mU l^{-1} (47-438 mU l^{-1})].

The index patient's younger brother (III-2, Family 2; Fig. 1a; Supplementary Table 2) was born at term by caesarian section after a pregnancy complicated by polyhydramnios with a birth weight of 3,230 g (-0.8 SDS). There were no neonatal problems. He also suffered from mild eczema early in life, and had uncomplicated chickenpox. He had mild speech delay, but otherwise normal developmental milestones. From the age of 2.3 years he demonstrated a similar growth pattern to his brother [height 97.3 cm (-3.1 SDS), weight 9.0 kg (-3.5 SDS)]. His endocrine evaluations resembled those of his brother. A glucagon GH provocation test showed a baseline GH of 4.2 ng ml^{-1} and peak GH of 13.9 ng ml^{-1}. An extended IGF1 generation test did not reach the normal IGF1 range (32 ng ml^{-1}, normal range: 52 - 297 ng ml^{-1}) when the highest rhGH dose was tested (Supplementary Table 1). IgE was increased 10-fold above normal (547 kU l^{-1}, normal: < 52 kU l^{-1}). At the age of 5.6 years, his height was 98.7 cm (-3.2 SD), weight 13.7 kg and HC, determined at 6.4 years, was 51.5 cm (-1.19 SDS). His IGF1 was < 25 ng ml^{-1} (52 - 297 ng ml^{-1}) and IGFBP3 1.00 mg l^{-1} (1.3-5.6 mg l^{-1}) with normal prolactin (143 mU l^{-1}, normal 47-358 mU l^{-1}). Bone age was severely delayed at 3 years.

Mother (II-2, Family 2; Fig. 1a) measured 155 cm (-1.5 SDS) with a HC of 54.8 cm (-0.45 SDS), had eczema as a child and was investigated for failure to thrive; father measured 172.5 cm (-0.8 SDS) and had eczema and hayfever. The mother had normal baseline endocrine investigations at the age of 33 years: prolactin 322 mU l^{-1}; basal GH, 0.3 ng ml^{-1}; IGF1, 113 ng ml^{-1} (normal, 69 - 268 ng ml^{-1}); and IGFBP3, 3.22 mg l^{-1}. Maternal aunt (II-3, Family 2) measured 155 cm (-1.5 SDS) and was diagnosed with celiac disease at the age of 3 years. Both mother and her sister (prolactin 1800 mU l^{-1}) also had a history of hyperprolactinemia. Maternal aunt was investigated at the age of 22 years. At that time she was underweight and on SSRI treatment for anxiety and depression. An MRI suggested a possible microadenoma, and she was started on Cabergoline for many years. When this was stopped, her prolactin had normalized and she was discharged from further follow up. Mother was also investigated at that time for hyperprolactinemia, including MRI of the pituitary, but was not started on treatment and prolactin normalized. Maternal grandfather (I-1, Family 2) suffered from eczema and was short-statured (157.5 cm, -3.0 SDS) as was maternal grandmother (149.9 cm, -2.3 SDS).

Targeted analysis of genomic DNA of both children revealed a heterozygous transition from cytosine to thymidine (c.1433C>T) within exon 12 of the *STAT5B* gene. The variant was inherited from the mother, who carries the variant as did her sister. The maternal grand-mother was carrier of the wild-type alleles; the maternal grand-father declined evaluation. The variant is predicted to result in an amino acid exchange from alanine to valine at the C-terminus of the STAT5B DNA-binding domain (p.Ala478Val).

Whole-exome sequencing was performed in both affected brothers, their parents, and their maternal aunt. As there were 2 affected brothers and a mother of milder short stature with a maternal grandfather with significant short stature, this pattern of inheritance could theoretically be consistent with a dominant mode of inheritance from the mother's side of the family, X-linked recessive inheritance, homozygous recessive or a compound heterozygous pattern. The analyses for the X-linked, homozygous recessive and compound heterozygous patterns of inheritance were identical to those in Family 1. No variants met any of these 3 criteria. A *de novo* analysis was performed looking for variants that were *de novo* in both siblings and, as expected, no variants were identified. This could only occur if germline mosaicism was present. Finally, we performed a dominant analysis searching for novel variants which were inherited from the mother, given the family history. There were thirteen variants that met these criteria (Supplementary Table 4). Of the 13 variants, 8 were predicted to be benign by either Polyphen2 or SIFT. Of the 5 remaining variants, 3 were present in the maternal aunt who had a similar degree of short stature to the mother. The c.1433C>T, p.Ala478Val variant in *STAT5B* was the lead biological candidate.

Family 3. Three of the 6 children born to non-consanguineous Caucasian parents presented with post-natal growth failure and proportionate microcephaly (head circumferences were between -3.0 to -4.2 SDS). The father had short stature (159.4 cm, -2.4 SDS) and the mother was of normal height (161.3 cm, -0.3 SDS); head circumferences were within normal ranges (54.7 cm, -0.28 SDS and 52.4 cm, -1.81 SDS, respectively). The female index case (Proband 3, Fig. 1a; subject II-1, Family 3), who was the oldest child, was born at term with a normal birth weight of 3,317 g and birth length of 48.2 cm

(Table 1). No growth data were available for the first few years of life. She was diagnosed with autoimmune thyroiditis at age 6.5 years with a fT4 of < 0.2 ng dl^{-1}, TSH of 341 µU ml^{-1}, and positive TPO antibodies. Despite normalization of TSH with L-T4 therapy, growth remained poor. At age 9.1 years, her height was 108.2 cm (-4.4 SDS) and weight 16.5 kg (-4.2 SDS). Basal GH was 1.99 ng ml^{-1}, stimulated GH (arginine/insulin tolerance test, AITT) peaked at 3.97 and 2.04 ng ml^{-1}. She had normal MRI of the brain and pituitary. GH therapy [0.30 mg/(kg*week)] was initiated at 9.1 years but was stopped after 9 months due to poor growth response. At chronological age 12.0 years, her bone age was 8.8 years (Greulich-Pyle method[3]), height 118.0 cm (-4.4 SDS) and weight 20.2 kg (-4.9 SDS). Serum IGF1 concentration was 137 ng ml^{-1} (normal: 104-456 ng ml^{-1}). rhGH therapy was re-started at 12.5 years [0.35 mg/(kg*week)]. At age 12.75 years, while on rhGH, serum IGF1 concentration was 208 ng ml^{-1} (-1.5 SDS), IGFBP3 was 3.8 mg l^{-1} (normal: 3.9-9.4mg l^{-1}) and acid labile subunit (IGFALS) was 13 mg l^{-1} (normal: 5.6-16.0). She was found to have mild pulmonic stenosis. Celiac disease was diagnosed (by positive serology and subsequent biopsy); a gluten-free diet was instituted. Growth of the index case modestly improved with puberty (Tanner stage B3P2 at age 14.3 years) and incremental increases in rhGH (to 0.7 mg/(kg*week). At age 14.9 years, while still on rhGH, she reached a height of 137.9 cm (-3.7 SDS) and weight of 29.6 kg (-4.7 SDS). She was switched to rhIGF1 therapy [titrated to 0.24 mg/(kg*day), twice daily doses] and, despite poor compliance, reached a height of 142.8 cm (-3.1 SDS) at age 15.9 (Tanner stage B5P4). Her prolactin concentrations were found to be elevated at 29.2 ng ml^{-1} (normal, < 18.0 ng ml^{-1}). It is of note that she did not have eczema and her serum IgE concentrations consistently measured within the normal range.

The affected sister of Proband 3 (subject II-2 of Family 3, Fig. 1a and Supplementary Table 2) was born at 39 weeks and had a normal birth weight of 3.289 kg. She presented with idiopathic short stature and at age 9.1 years, had a height of 117 cm (-3.3 SDS) and weight of 20 kg (-3.1 SDS). rhGH therapy (titrated to 0.49 mg/(kg*week) was initiated and sustained until age 13.8 years. Serum IGF1 concentrations while on rhGH were within the normal range (171 ng ml^{-1}, -1.2 SDS, age 10.6 years). At age 11.2 years, her bone age was 8.8 years, height was 127 cm (-2.7 SDS) and weight 24.7 kg (-2.5 SDS). She had Tanner stage B3P1 at age 12.2 years, and at age 13.2 years, still on rhGH, height was 140 cm (-2.5 SDS). Eczema was noted, and she had confirmed elevated serum IgE, with a recent measurement of 1,133 kU l^{-1} (normal, ≤ 629 kU l^{-1}). Serum prolactin concentrations were normal (10.2 ng ml^{-1}; normal, < 18.0).

The affected brother of Proband 3 (subject II-5 of Family 3, Fig. 1a and Supplementary Table 2), similar to the affected sister, presented with idiopathic short stature, eczema and elevated serum IgE (751 kU l^{-1}; normal, ≤ 307 kU l^{-1}). At chronological age 3.6 years, he had a bone age of 2.75 years, height of 87 cm (-3.2 SDS) and weight of 10.6 kg (-3.8 SDS). At that time serum IGF1 was below normal at 21 ng ml^{-1} (-2.6 SDS) and prolactin was slightly above the reference range (11.2 ng ml^{-1}; normal, < 10.0 ng ml^{-1}) at 6.1 years. At age 5.75 years, when height was 98.8 cm (-3.0 SDS), rhIGF1 (0.24 mg/(kg*day) was initiated, but was suspended at age 6.7 years (height 105.1 cm; -2.7 SDS) due to intolerance of injections and thus questionable compliance.

The youngest brother (2.5 years), subject II-6 (Fig. 1a and Supplementary Table 2), who had elevated IgE values (354 and 492 kU l^{-1}; > 2.0 SD), had normal height for age (-0.4 SDS at 3.5 years) with a HC of -1.02 SDS. Because of his young age close monitoring of height to detect growth deviation is ongoing. The remaining 3 siblings were of normal stature, with unremarkable biochemistries. Interestingly, the short-statured father also had an elevated IgE of 264 kU l^{-1} (> 2.0 SD) while the mother had IgE of < 2 kU l^{-1}.

Exome sequencing was performed on the father, three affected siblings (II-1, II-2, and II-5) as well as one of the unaffected brothers (II-3). As three of the children had significant short stature as did the father, we therefore assumed that there was a dominant form of inheritance. We included nonsynonymous variants that were heterozygous in the three affected siblings and their father and absent in the unaffected brother. Five variants met these criteria (Supplementary Table 4). One of the five variants was predicted to be benign. Of the four remaining genes, *MARCO* and *TPP2* had no clear connection to growth or the growth hormone/IGF-I axis. *STAT5B* and *JAK2* were both candidate variants. Variant segregation pattern in the family indicated that the *JAK2* variant was present in the father, 3 affected siblings as well as two of the brothers without short stature (II-4 and II-6). The novel *JAK2* variant (exon 18, NM_001322194.1: c.2374C>T, p.Pro792Ser) is located within the JH2 ("pseudokinase") domain where a number of somatic and germline dominantly inherited gain-of-function JAK2 variants associated with various hematologic disorders (thrombocythemia-3, thrombocytopenia, polycythemia vera) have been described. The lack of symptoms indicative of hematologic abnormalities in Family 3, and the normal growth (height SDS of -1.1 at age 7.9 years; IGF1 serum concentration, 153 ng ml^{-1}, -0.2 SDS) and health (lack of eczema; IgE of 9.7 kU l^{-1}; mean, 18 kU l^{-1}, Mayo Clinic) of subject II-4 (who had wild-type STAT5B), suggest the heterozygous JAK2 p.Pro792Ser is unlikely to be causal of the clinical symptoms observed in Family 3.

The *STAT5B* variant was present in the father, 3 affected siblings and the youngest brother without short stature (II-6). Subject II-6 was still quite young at the time of evaluation and it was felt that he may not yet have manifested the growth failure. Additionally, as his IgE concentrations were elevated, we concluded that he was clinically affected and thus only the *STAT5B* variant segregated with clinical affectation status in the family. The c.1421A>G transition in exon 12 of the *STAT5B* gene is predicted to result in an arginine for glutamine substitution (p.Gln474Arg). Confirmation of the heterozygous *STAT5B* c.1421A>G variant and segregation analysis in all 6 children and their parents was performed by Sanger sequencing.

For family 3, we performed a secondary analysis of the exome data to investigate whether there was a recessive gene contributing to microcephaly in this family. Specifically, we considered that individuals II-1, II-2, and II-5 all were affected with microcephaly and the father and brother (II-3) who were whole-exome sequenced were not affected. We performed three types of recessive analysis: homozygous recessive, compound heterozygous, and X-linked. For all analyses, we only included nonsynonymous variants with a minor allele frequency below 1% in the ExAC and 1000 Genomes databases as well as in our internal sequencing center database. For the homozygous recessive analysis, we searched for variants that were homozygous in the 3 affected siblings, heterozygous in the father, and either wild-type

or heterozygous in the brother. For the X-linked, we look for variants that were hemizygous in the 3 affected siblings and wild-type in the father and brother. For the compound heterozygous analysis, we looked for genes in which there were 2 or more heterozygous variants shared by the 3 affected siblings and the father was heterozygous for one of the variants and wild-type for the other (i.e. the variants were inherited in trans) and the brother did not carry both variants. There were no variants/genes that met these analytic criteria for any one of the three recessive inheritance patterns. Therefore, while we cannot conclusively rule out the possibility of an additional genetic variant contributing to the microcephaly seen in family 3, there are no clear recessive candidates seen in the available exome data.

Supplementary Table 1. Extended IGF1 generation test in affected siblings of Family 2[a]

patient	step 1 [0.7 mg/(m²*d)] [b]		step 2 [1.4 mg/(m²*d)]		step 3 [2.8 mg/(m²*d)]		ref. range
	day 1	day 14	day 1	day 14	day 1	day 14	
III-1 (index)							
IGF1 (ng ml^{-1})	30	56	44	61	28	83	57 - 316
IGFBP3 (mg l^{-1})	1.89	2.84	2.62	2.87	2.18	3.33	1.4 - 6.1
III-2 (brother)							
IGF1 (ng ml^{-1})	<25	<25	<25	<25	<25	32	52 - 297
IGFBP3 (mg l^{-1})	1.04	1.42	1.17	1.64	1.14	2.37	1.3 - 5.6

[a]) performed at 5 and 2.9 years (index and brother, respectively) as described in Online Methods[2]; [b]) rhGH dose

Supplementary Table 2. Penetrance of major clinical characteristics of autosomal dominant STAT5B deficient patients and affected family members

Subjects [a]	age	height (cm) [SDS]	RC [b]	HC (age:cm) [SDS]	RC	IGF1 (ng/ml) [SDS or *ref. range*]	RC	IgE (kU l^{-1}) [SDS or *ref. range*]	RC
Family 1 (p.Gln177Pro)									
index, male (II-2)	14.5	131.5 [-5.3]	↓	21y:56 [-0.53]	→	56 [*76 - 499*]	↓	156 / 340 [c] [< *200* / < *114*]	→ / ↑
twin brother (II-3)	14.5	131.5 [-5.3]	↓	21y:55 [-1.1]	→	58 [*76 - 499*]	↓	228 / 382 [c] [< *200* / < *114*]	↑ / ↑
Family 2 (p.Ala478Val)									
index, male (III-1)	1.8	76.8 [-2.9]	↓	9.4y:51.6 [-1.7]	→	< 25 [*51 - 303*]	↓	118 [< *52*]	↑
brother (III-2)	2.3	79.3 [-3.1]	↓	6.4y:51.5 [-1.2]	→	< 25 [*52 - 297*]	↓	547 [< *52*]	↑
mother (II-2)	33	155.0 [-1.5]	→	36y:54.8 [-0.4]	→	113 [*69 - 268*]	→	nd	-
mat. aunt (II-3)	30	155.0 [-1.5]	→	nd	-	nd	-	nd	-
Family 3 (p.Gln474Arg)									
index, female (II-1) [d]	12.8	123.8 [-4.5]	↓	12.8y:48.8 [-3.7]	↓	208 [-1.5]	→	28 / 127 [c] [< *114* / < *629*]	→ / →
sister (II-2) [d]	12.6	139.5 [-2.2]	↓	10.6y:48.5 [-3.2]	↓	322 [-0.4]	→	598 / 1133 [c] [> 2.0 / < *629*]	↑ / ↑
brother (II-5)	5.6	98.3 [-2.9]	↓	5.6y:47.2 [-3.0]	↓	34 [-1.8]	→	261 / 751 [c] [> 2.0 / < *307*]	↑ / ↑
brother (II-6)	2.5	89.3 [-0.4]	→	2.0y:47.2 [-1.0]	→	34 [-0.7]	→	354 / 492 [c] [> 2.0]	↑ / ↑
father (I-1)	39	159.4 [-2.4]	↓	39y:54.7 [-0.3]	→	128 [*83 - 233*]	→	264 [< *180*]	↑
Penetrance			**↓ 8/11**		**↓ 3/10**		**↓ 4/10**		**↑ ~ 8/9**

nd, not determined; [a]) designation of subjects refers to pedigrees in Fig. 1a (main text); [b]) RC, comparison to reference; [c]) measured at 2 occasions; [d]) patient on rhGH treatment

***Supplementary Table 3*.** Compound heterozygous candidate variants identified by WES analysis in family 1.

Chr:Position	Ref	Alt	dbSNP ID	Gene	Protein Effect	ExAC (MAF)	PolyPhen-2 Raw Score	SIFT Raw Score
3:8665261	C	T	NP	SSUH2	Ala319Thr	NP	0.045	0.153
3:8672534	T	C	rs146804066	SSUH2	Glu161Gly	0.0017	0.436	0.097
3:49680130	G	A	rs148125466	BSN	Ala355Thr	0.0034	0.901	0.002
3:49698075	G	A	rs140228596	BSN	Val2933Ile	0.0015	0.001	0.22
11:6568564	C	T	rs201009127	DNHD1	Thr2132Ile	0.0003	0.383	0.029
11:6585767	C	T	NP	DNHD1	Pro3497Ser	NP	0.005	0.029

NP, not present; Chr, chromosome; Position: build hg19; Ref, reference allele; Alt, alternate allele; MAF, minor allele frequency. PolyPhen-2 scores (HumVar) range from 0.0 (benign) to 1.0 (damaging). SIFT score ranges from 0 to 1. The amino acid substitution is predicted damaging if the score is <= 0.05, and tolerated if the score is > 0.05.

***Supplementary Table 4*.** Dominant candidate variants identified by WES analysis in families 2 and 3.

Chr:Position	Ref	Alt	Present in Aunt	Gene	Protein Effect	PolyPhen-2 Raw Score	SIFT Raw Score
Family 2							
1:28209083	C	G	Yes	THEMIS2	Ser416Arg	0.001	0.018
2:63282892	G	A	No	OTX1	Ser169Asn	0.483	0.191
2:160637502	G	C	No	LY75-CD302	Asp1703Glu	0.968	0.192
2:198270154	C	T	Yes	SF3B1	Ala428Thr	0.213	0.061
3:196234961	T	G	Yes	SMCO1	Asn148His	0.003	0.218
4:126402718	G	T	No	FAT4	Arg4214Leu	0.269	0.588
10:89473012	T	C	No	PAPSS2	Leu109Pro	1	0
11:64884498	G	A	No	ZNHIT2	Leu210Phe	0.996	0
14:24710283	T	C	Yes	TINF2	Ile183Val	0.001	0.535
14:75366659	G	A	Yes	DLST	Arg312Lys	0.944	0.028
17:18164454	G	A	Yes	MIEF2	Gly2Glu	N/A	0
17:37421685	G	C	Yes	FBXL20	Gln319Glu	0.743	0.258
17:40368072	G	A	Yes	STAT5B	Ala478Val	0.958	0.001
Family 3							
2:119729073	AG	A	NA	MARCO	Met143CysfsTer121	NA	NA
9:5080623	C	T	NA	JAK2	Pro792Ser	1	0.014
9:6015191	T	G	NA	RANBP6	Glu139Asp	0.029	0.183
13:103271164	C	A	NA	TPP2	Asp196Glu	0.984	0
17:40368084	T	C	NA	STAT5B	Gln474Arg	0.921	0

NA, not applicable; Chr, chromosome; Position: build hg19; Ref, reference allele; Alt, alternate allele; MAF, minor allele frequency. PolyPhen-2 scores (HumVar) range from 0.0 (benign) to 1.0 (damaging). SIFT score ranges from 0 to 1. The amino acid substitution is predicted damaging if the score is <= 0.05, and tolerated if the score is > 0.05.

Supplementary Table 5. *in silico* analysis of STAT5B variants

patient	position			MAF [d]	prediction [e]		
	genomic [a]	coding [exon] [b]	protein [c]		PP-2	SIFT	Mut.-Taster
1	g.40375420T>G	c.530A>C [5]	p.(Gln177Pro)	-/-/-	P	P	P
2	g.40368072G>A	c.1433C>T [12]	p.(Ala478Val)	-/-/-	P	P	P
3	g.40368084T>C	c.1421A>G [12]	p.(Gln474Arg)	-/-/-	P	P	P

[a]) GRCh37 (hg19), NC_000017.10; [b]) NM_012448.3; [c]) UniProtKB P51692; [d]) Exome Aggregation Consortium (ExAC) / Exome Sequencing Project (ESP) / 1000 Genomes Project, all accessed through UCSC Genome Browser [http://genome.ucsc.edu]; [e]) P, predicted to be pathogenic, PP-2 (Polyphen-2 [http://genetics.bwh.harvard.edu/pph2/index.shtml])[4], SIFT [http://sift.jcvi.org/www/SIFT_BLink_submit.html][5], Mut.-Taster (MutationTaster [http://www.mutationtaster.org/])[6]; all *in silico* analysis performed September 2016.

Supplementary Table 6. Concordance rates of single nucleotide variants between family members in family 1

Father	Proband 1	55.37%
Father	Proband 2	55.37%
Mother	Proband 1	54.08%
Mother	Proband 2	54.13%
Proband 1	Proband 2	98.43%

Supplementary Figure 1.

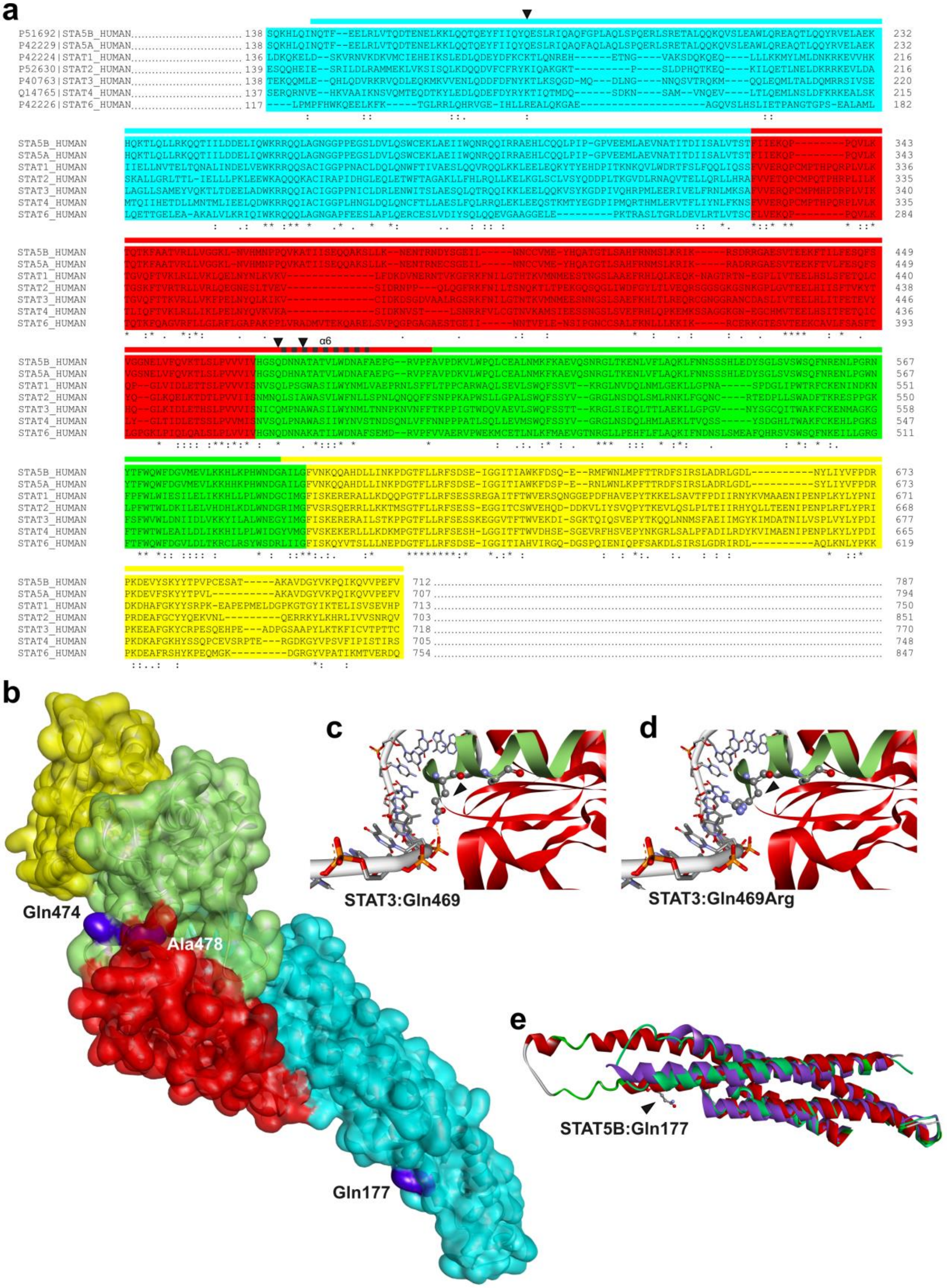

Supplementary Figure 1. The autosomal-dominant STAT5B variants replace conserved amino acids in structural motifs necessary for proper STAT function. (**a**) Alignment of all human STAT paralogues (Clustal Omega [http://www.ebi.ac.uk/Tools/msa/clustalo/])[7]. Only the portion of the full-length alignment corresponding to the sequence included in the 3D STAT5B model (b) is shown and colored accordingly (cyan, four-helix bundle, CCD; red, eight-stranded β-barrel, DBD; green, α-helical linker domain; yellow, SH2 domain); arrow heads, variant STAT5B positions; consensus symbols: asterisk, identical positions; colon, strongly similar positions; period, weakly similar positions. Note that domain assignment corresponds to the boundaries published for STAT5A[8] and STAT3[9] that slightly differ from functionally defined boundaries, in which α-helix 6 (α6; hatched bold line) is part of the DNA-binding domain[10–12] and that were used throughout the article (bold line and Fig. 1b). (**b**) Model of STAT5B tertiary structure computed by SwissModel [https://swissmodel.expasy.org/][13] using mouse STAT5A as template (pdb:1Y1U)[8]. Color scheme of the semi-transparent surface model as in (a); blue spheres, wild-type residues of the variant amino acids; note surface exposure of Gln177 and Gln474 (deep blue), whereas Ala478 (faint blue) is buried within the first α-helix (α6) of the linker domain. (**c,d**) Simulation of the p.Gln469Arg substitution in the 3D backbone (shown as flat ribbon diagram; residues starting from Lys488 omitted for visualization purposes) of mouse STAT3 (pdb:1BG1)[9] in complex with DNA (stick-tube diagram; no three-dimensional structure of STAT5:DNA complex available). Replacement of Arg for Gln (amino acid shown as ball and stick) performed by Modeller[14] (accessed through Structuropedia [http://structuropedia.org/]) is likely to interfere with interactions between the loop connecting the β-barrel and linker domain and nucleotides of the major groove as demonstrated by the loss of a H-bond (yellow, dashed line). (**e**) Gln177 (shown as ball and stick) resides at the C-terminal end of CCD α-helix 1 (ribbon diagram) of STAT5B (red; modelled as described in (b)) adjacent to the extended α1-α2 bend that significantly differs from the analogous structures in STAT1 (pdb:1BF5, blue)[10] and STAT3 (pdb:3CWG, green)[15]. Superimposition performed using 3d-SS [http://cluster.physics.iisc.ernet.in/3dss/][16]. All diagrams (b-e) generated using Discovery Studio v3 (Biovia, San Diego, CA).

Supplementary Figure 2.

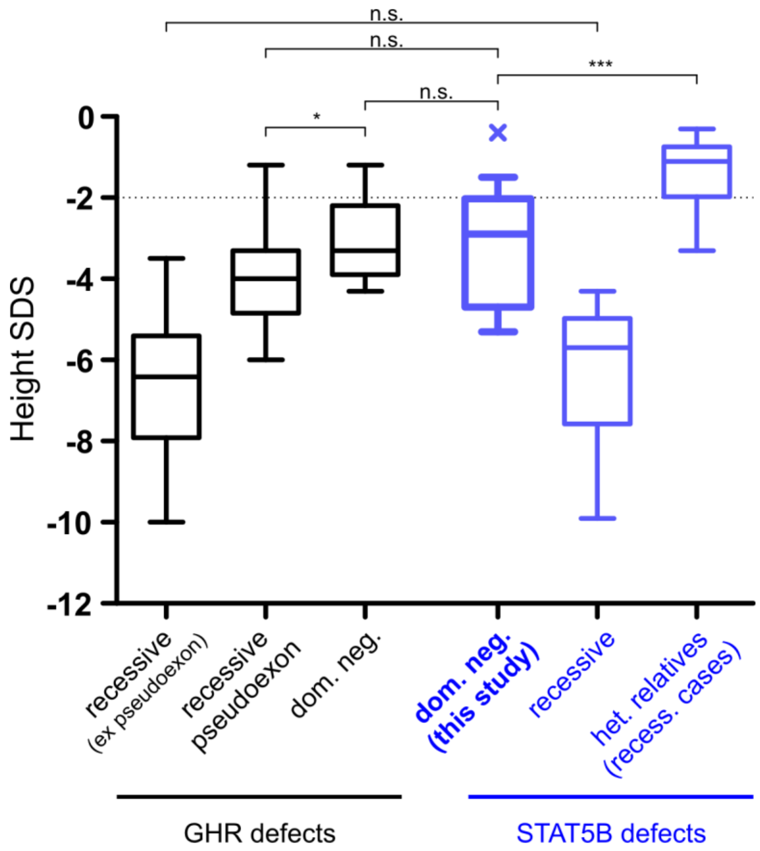

Supplementary Figure 2. Carriers of dominant-negative STAT5B mutations have similar height deficits as patients carrying dominant-negative GHR mutations. Height SDS values of GHIS patients with GHR mutations (black) were compared with height SDS values of STAT5B mutational carriers (blue; dominant-negative, this study, n=11; autosomal-recessive[12,17–22], n=10; heterozygous, not affected relatives of patients with recessive mutations[23], n=14). GHR patients were grouped according to type of mutation: autosomal-dominant (dom. neg.; n=16), autosomal-recessive missense and protein-truncating variants (recessive ex pseudoexon; n=100), autosomal-recessive pseudoexon 6 activation mutation (c.618+792A>G; n=21; leading to in-frame insertion of 36 amino acids and a GHR protein with residual activity[24]). Box (median, 25th and 75th percentiles) and whiskers (minimum and maximum values) plots; statistical analysis by Student's t-test, * $P < 0.05$, ** $P < 0.01$, *** $P < 0.001$, P value not shown: $P < 0.0001$; in the group of patients with dominant-negative STAT5B mutations subject II-6, family 3 (blue cross) was excluded from analysis due to his young age (if included, the difference between the STAT5b dominant-negative group versus STAT5B heterozygous relatives group remains significant, $P = 0.0053$).

Supplementary Figure 3.

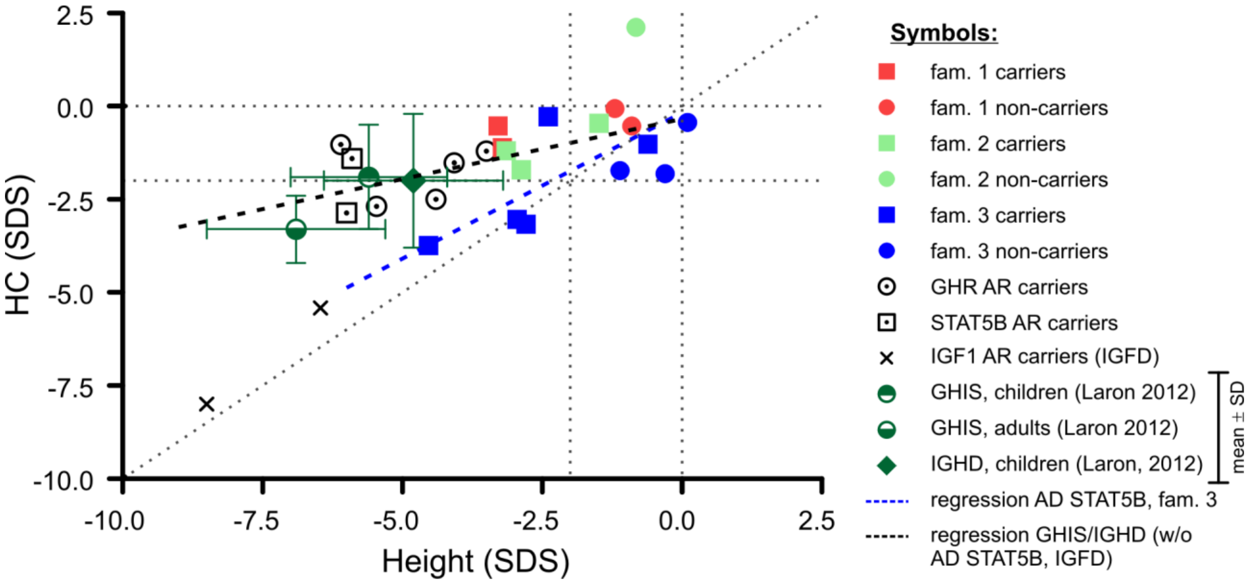

Supplementary Figure 3. Microcephaly is a feature of some but not all AD *STAT5B* mutation carriers of family 3. Subjects II-1, II-2 and II-5 of family 3 present with microcephaly with a head circumference (HC)-height ratio that closely resembles that of carriers of AR *IGF1* mutations (IGFD)[25,26]. In contrast, the HC-height ratio of mutation carriers of families 1 and 2 is in line with the pattern typically observed in conditions of isolated GH deficiency (IGHD)[27] and GHIS caused by GHR[27–31] or AR STAT5B[17,32] mutations. In these conditions microcephaly is only notable in patients with severe short stature. For patients with IGHD (IGHD, children: n=12) and subsets of patients with GHIS (GHIS, children: n=12 and GHIS, adults: n=22) data were taken as published by Laron et al., 2012[27] and plotted as mean ± S.D.

Supplementary Figure 4.

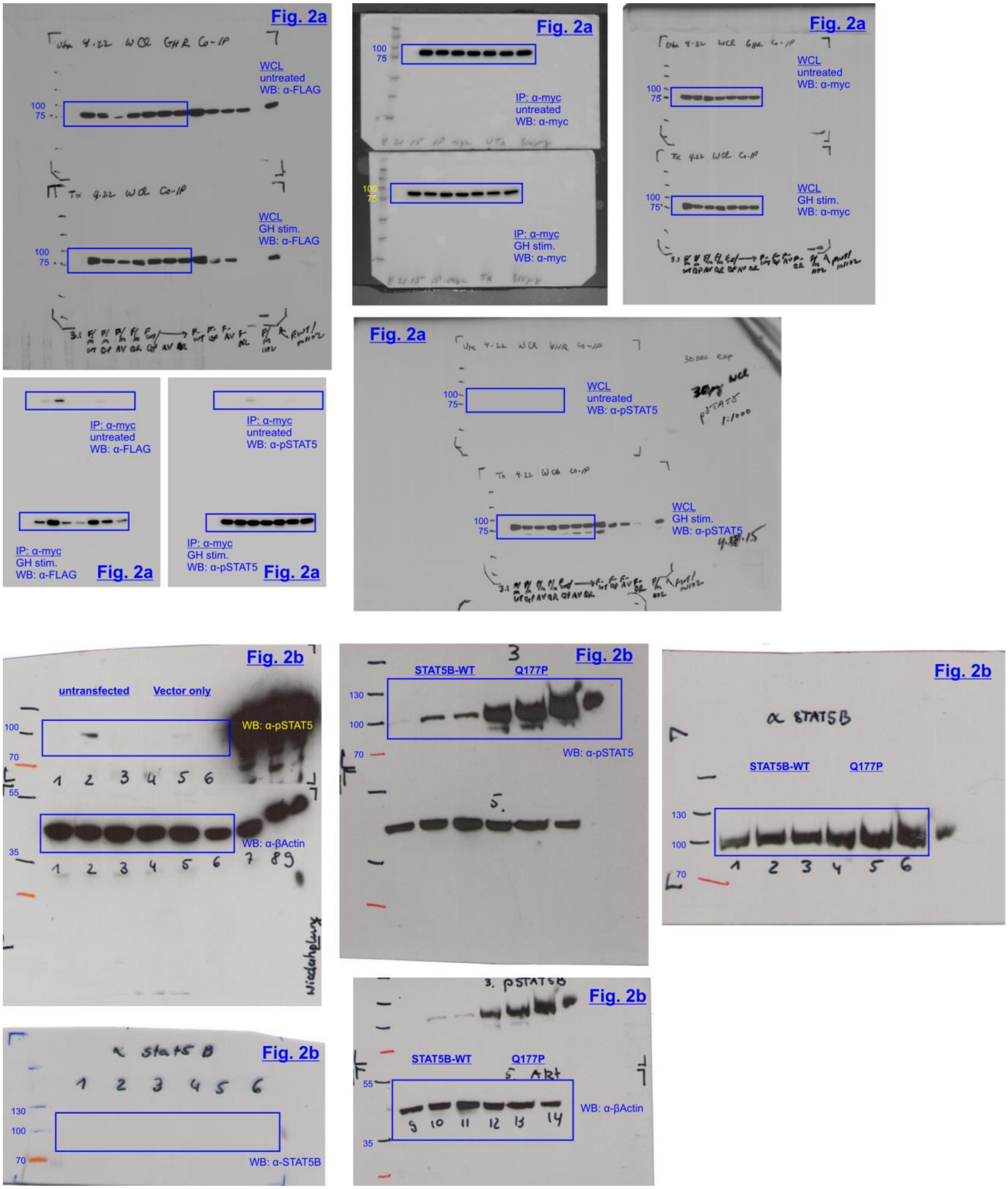

Supplementary Figure 4. Uncropped captures of immunoblots (Fig. 2a and 2b). Cropped areas are marked by blue boxes and the figures and panels in which they appear are indicated. WCL, whole cell lysates; WB, antibody used for immunoblotting; IP, antibody used for immunoprecipitation.

Supplementary Figure 5.

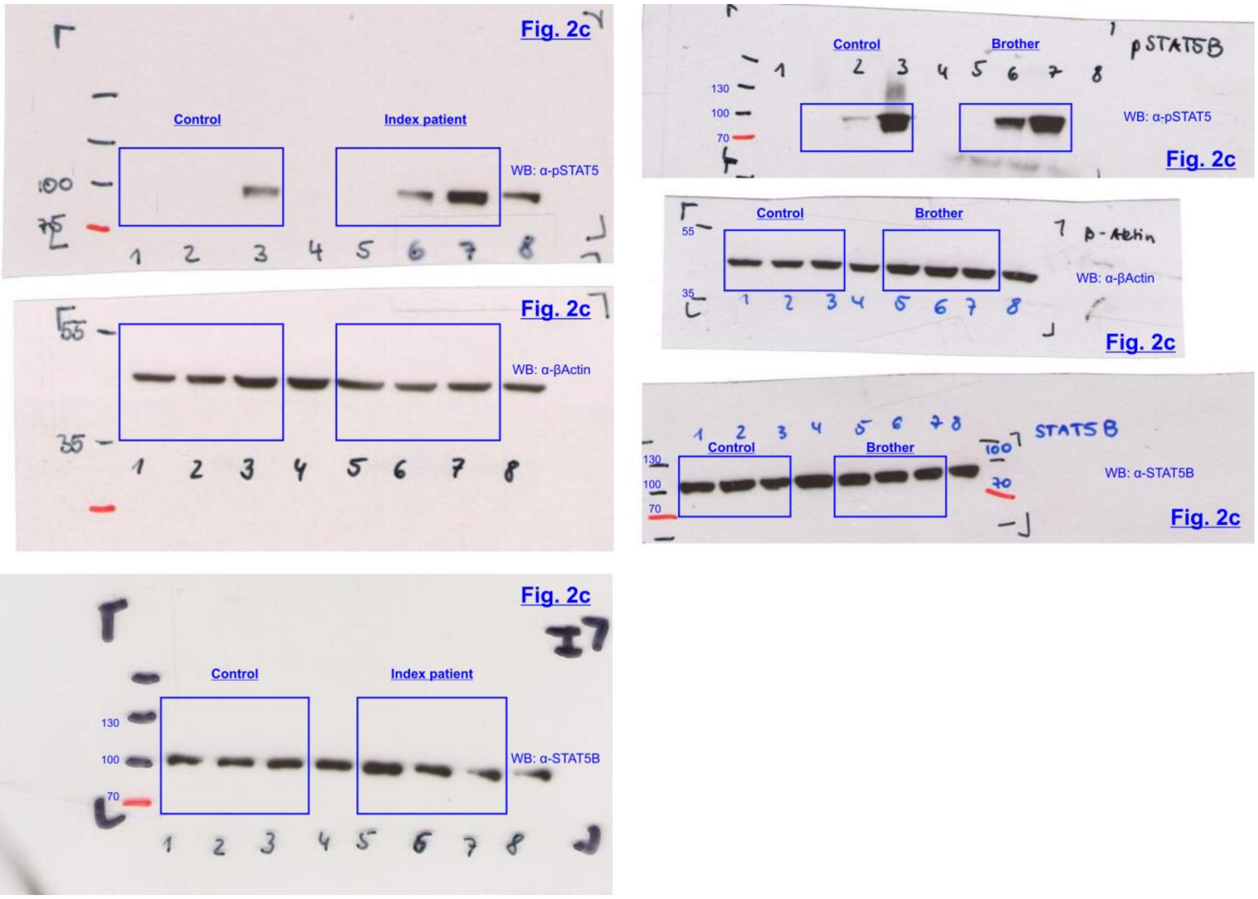

Supplementary Figure 5. Uncropped captures of immunoblots (Fig. 2c). Cropped areas are marked by blue boxes and the figures and panel in which they appear are indicated. WB, antibody used for immunoblotting.

Supplementary References

1. Tanner, J.M., Healy, M.J.R., Goldstein, H. & Cameron, N. *Assessment of Skeletal Maturity and Prediction of Adult Height (TW3) Method.* 3rd ed. (Saunders Ltd, 2001).
2. Buckway, C.K., Guevara-Aguirre, J., Pratt, K.L., Burren, C.P. & Rosenfeld, R.G. The IGF-I generation test revisited: a marker of GH sensitivity. *J. Clin. Endocrinol. Metab.* **86,** 5176–5183 (2001).
3. Greulich, W.W. & Pyle, S.I. *Radiographic atlas of skeletal development of the hand and wrist.* 2nd ed. (Stanford Univ. Press, Stanford, Calif., 1959).
4. Adzhubei, I.A. *et al.* A method and server for predicting damaging missense mutations. *Nat. Methods* **7,** 248–249 (2010).
5. Ng, P.C. & Henikoff, S. Predicting deleterious amino acid substitutions. *Genome Res.* **11,** 863–874 (2001).
6. Schwarz, J.M., Cooper, D.N., Schuelke, M. & Seelow, D. MutationTaster2: mutation prediction for the deep-sequencing age. *Nat. Methods* **11,** 361–362 (2014).
7. Sievers, F. *et al.* Fast, scalable generation of high-quality protein multiple sequence alignments using Clustal Omega. *Mol. Syst. Biol.* **7,** 539 (2011).
8. Neculai, D. *et al.* Structure of the unphosphorylated STAT5a dimer. *J. Biol. Chem.* **280,** 40782–40787 (2005).
9. Becker, S., Groner, B. & Muller, C.W. Three-dimensional structure of the Stat3beta homodimer bound to DNA. *Nature* **394,** 145–151 (1998).
10. Chen, X. *et al.* Crystal structure of a tyrosine phosphorylated STAT-1 dimer bound to DNA. *Cell* **93,** 827–839 (1998).
11. Li, J. *et al.* Structural basis for DNA recognition by STAT6. *Proc. Natl. Acad. Sci. U.S.A.* **113,** 13015–13020 (2016).
12. Hwa, V., Nadeau, K., Wit, J.M. & Rosenfeld, R.G. STAT5b deficiency: lessons from STAT5b gene mutations. *Best Pract. Res. Clin. Endocrinol. Metab.* **25,** 61–75 (2011).
13. Biasini, M. *et al.* SWISS-MODEL: modelling protein tertiary and quaternary structure using evolutionary information. *Nucleic Acids Res.* **42,** W252-W258 (2014).
14. Webb, B. & Sali, A. Comparative Protein Structure Modeling Using MODELLER. *Curr. Protoc. Bioinformatics* **54,** 5.6.1-5.6.37 (2016).
15. Ren, Z. *et al.* Crystal structure of unphosphorylated STAT3 core fragment. *Biochem. Biophys. Res. Commun.* **374,** 1–5 (2008).
16. Sumathi, K., Ananthalakshmi, P., Roshan, M. N. A. Md. & Sekar, K. 3dSS: 3D structural superposition. *Nucleic Acids Res.* **34,** W128-W132 (2006).
17. Scaglia, P.A. *et al.* A novel missense mutation in the SH2 domain of the STAT5B gene results in a transcriptionally inactive STAT5b associated with severe IGF-I deficiency, immune dysfunction, and lack of pulmonary disease. *J. Clin. Endocrinol. Metab.* **97,** E830-839 (2012).
18. Kofoed, E.M. *et al.* Growth hormone insensitivity associated with a STAT5b mutation. *N. Engl. J. Med.* **349,** 1139–1147 (2003).
19. Hwa, V. *et al.* Growth Hormone Insensitivity and Severe Short Stature in Siblings: A Novel Mutation at the Exon 13-Intron 13 Junction of the STAT5b Gene. *Horm. Res. Paediatr.* **68,** 218–224 (2007).

20. Pugliese-Pires, P.N. *et al.* A novel STAT5B mutation causing GH insensitivity syndrome associated with hyperprolactinemia and immune dysfunction in two male siblings. *Eur. J. Endocrinol.* **163,** 349–355 (2010).
21. Bernasconi, A. *et al.* Characterization of immunodeficiency in a patient with growth hormone insensitivity secondary to a novel STAT5b gene mutation. *Pediatrics* **118,** 92 (2006).
22. Hwa, V. *et al.* Severe growth hormone insensitivity resulting from total absence of signal transducer and activator of transcription 5b. *J. Clin. Endocrinol. Metab.* **90,** 4260–4266 (2005).
23. Scalco, R.C. *et al.* STAT5B mutations in heterozygous state have negative impact on height: another clue in human stature heritability. *Eur. J. Endocrinol.* **173,** 291–296 (2015).
24. Maamra, M. *et al.* A 36 residues insertion in the dimerization domain of the growth hormone receptor results in defective trafficking rather than impaired signaling. *J. Endocrinol.* **188,** 251–261 (2006).
25. Walenkamp, M.J. *et al.* Homozygous and heterozygous expression of a novel insulin-like growth factor-I mutation. *J. Clin. Endocrinol. Metab.* **90,** 2855–2864 (2005).
26. Woods, K.A., Camacho-Hubner, C., Savage, M.O. & Clark, A.J. Intrauterine growth retardation and postnatal growth failure associated with deletion of the insulin-like growth factor I gene. *N. Engl. J. Med.* **335,** 1363–1367 (1996).
27. Laron, Z., Iluz, M. & Kauli, R. Head circumference in untreated and IGF-I treated patients with Laron syndrome: comparison with untreated and hGH-treated children with isolated growth hormone deficiency. *Growth Horm. IGF Res.* **22,** 49–52 (2012).
28. Jorge, A.A.L., Souza, Silvia C. A. L., Arnhold, I.J.P. & Mendonca, B.B. The first homozygous mutation (S226I) in the highly-conserved WSXWS-like motif of the GH receptor causing Laron syndrome: supression of GH secretion by GnRH analogue therapy not restored by dihydrotestosterone administration. *Clin. Endocrinol. (Oxf)* **60,** 36–40 (2004).
29. Walker, J.L. *et al.* A Novel Mutation Affecting the Interdomain Link Region of the Growth Hormone Receptor in a Vietnamese Girl, and Response to Long-Term Treatment with Recombinant Human Insulin-Like Growth Factor-I and Luteinizing Hormone-Releasing Hormone Analogue. *J. Clin. Endocrinol. Metab.* **83,** 2554–2561 (1998).
30. Walenkamp, M.J.E. *et al.* Genetic analysis of GHR should contain sequencing of all coding exons and specific intron sequences, and screening for exon deletions. *Horm. Res. Paediatr.* **80,** 406–412 (2013).
31. Aisenberg, J. *et al.* Atypical GH Insensitivity Syndrome and Severe Insulin-Like Growth Factor-I Deficiency Resulting from Compound Heterozygous Mutations of the GH Receptor, Including a Novel Frameshift Mutation Affecting the Intracellular Domain. *Horm. Res. Paediatr.* **74,** 406–411 (2010).
32. Vidarsdottir, S. *et al.* Clinical and biochemical characteristics of a male patient with a novel homozygous STAT5b mutation. *J. Clin. Endocrinol. Metab.* **91,** 3482–3485 (2006).
