# Supplementary material for: Dominant-negative STAT5B mutations cause growth hormone insensitivity with short stature and mild immune dysregulation
Source: Nat Commun. 2018 May 29;9:2105. doi: 10.1038/s41467-018-04521-0 (PMC5974024; doi:10.1038/s41467-018-04521-0)
Supplement: Supplementary file 3 — Description of Additional Supplementary Files [file 41467_2018_4521_MOESM3_ESM.pdf]

# Description of Additional Supplementary Files

File Name: Supplementary Data 1

Description: Immunological profiles of index patients and affected relatives
